# Social Networking of Group Two Innate Lymphoid Cells in Allergy and Asthma

**DOI:** 10.3389/fimmu.2018.02694

**Published:** 2018-11-20

**Authors:** Benjamin P. Hurrell, Pedram Shafiei Jahani, Omid Akbari

**Affiliations:** Department of Molecular Microbiology and Immunology, Keck School of Medicine, University of Southern California, Los Angeles, CA, United States

**Keywords:** ILC2, allergic disease, asthma, activation, inhibition

## Abstract

Allergic diseases including asthma, chronic rhinosinusitis, and atopic dermatitis are common conditions worldwide. While type 2 immune responses induced by T-cells significantly cause allergic inflammation, the recently identified group two innate lymphoid cells (ILC2s) are emerging as critical players in the development of allergy. Upon allergen exposure, ILC2s are rapidly activated by cytokines released by epithelial cells. Activated ILC2s release various effector cytokines altogether contributing to the pathogenesis of allergy and can even cause inflammation in the absence of T-cells, as observed in asthma. Although the factors inducing ILC2 activation have been identified, evidence suggests that multiple factors can enhance or repress ILC2 proliferation, trafficking, or secretion of effector cytokines upon allergic inflammation. In this review, we discuss the recent findings that influence ILC2 activation and the resulting effects on the pathogenesis of allergy. A better understanding of how ILC2s are modulated will open the door to the development of new therapeutic strategies against allergic diseases.

## Introduction

Allergic diseases are highly diverse and common conditions caused by the inappropriate sensitization of the immune system to environmental antigens. Upon re-exposure to these allergens, sensitized individuals develop allergic symptoms including rash, wheezing, and shortness of breath. Most inflammatory responses seen in allergic diseases are caused by the release of type 2 cytokines from activated T helper 2 (Th2) cells. Main features of allergic reactions include smooth muscle cell contraction, mucus production, release of IgE, increased vascular permeability and recruitment of effector cells including eosinophils, basophils, and mast cells (1). However, type 2 cytokines are not only produced by Th2 cells, with Th9 (2), follicular T helper cells (3, 4) and inflammatory cells further contributing to type 2 cytokine secretion. Invariant natural killer T-cells also produce large amounts of cytokines and induce airway inflammation independent of T-cells (5). Recently, group 2 innate lymphoid cells (ILC2s) were described as a source of cytokines during allergic inflammation.

ILC2s are a subset of the innate lymphoid cells family described in three independent studies (6–8), following pioneer work in the early 2000s (9). Mouse and human ILC2s are phenotypically comparable, lineage negative, non T-, non B-lymphocytes (Table 1). Unlike T-cells, ILC2s lack antigen specific markers and instead are rapidly activated by alarmins released following tissue damage, pathogen recognition or allergen challenge. Activated ILC2s release high amounts of type 2 cytokines and contribute to a growing number of human diseases (10, 11) including **chronic rhinosinusitis with nasal polyps (CRSwNP)** (12–17) and **atopic dermatitis (AD)** (18, 19), two common allergic diseases of the paranasal sinuses (upper airways) and skin, respectively. Several studies have described the involvement of ILC2s in human **allergic asthma** (20–24). Asthma is a common, heterogeneous chronic inflammatory disease of the lower airways characterized by airway hyperreactivity (AHR) and reversible bronchoconstriction. Patients with asthma have a greater number of total and activated blood ILC2s compared to healthy controls (20), with increased numbers of ILC2s further detected in bronchioalveolar lavage (BAL) fluids of asthmatics (21). In line with this, ILC2s in blood and sputum are increased in severe compared to mild asthma patients (24), and in the sputum of children with severe asthma (23). Interestingly, increased ILC2 numbers are correlated with increased eosinophilia (22, 24). Altogether, these findings suggest that ILC2s are critical in human asthma.

**Table 1:** Mouse and human ILC2 markers.

**Biomarkers**	**Mouse**	**Human**
CD45	+	+
CD90 (Thy1)	+	–
CD25 (IL-2Ra)	+	+
CD127 (IL-7Ra)	+	+
ST2 (IL-33R)	+	+
IL17Rb (IL-25R)	+	+
CD161 (NKR-P1A)	–	+
CD278 (ICOS)	+	+
CD294 (CRTH2)	+	+
KLRG1	+	+
CD117 (c-kit)	+	+
Sca-1	+	–
CD194 (CCR4)	–	+
CD44	+	–
Mouse lineage negative	CD3, B220, Gr-1, CD11b, CD11c, Ter119, NK1.1, TCR-γδ, FCεRI, Mac-1	
Human lineage negative	CD1a, CD3, CD14, CD16, CD19, CD20, CD56, CD123, CD235a, CD11b, FCεRI, TCR-δ	

**Figure 1:**
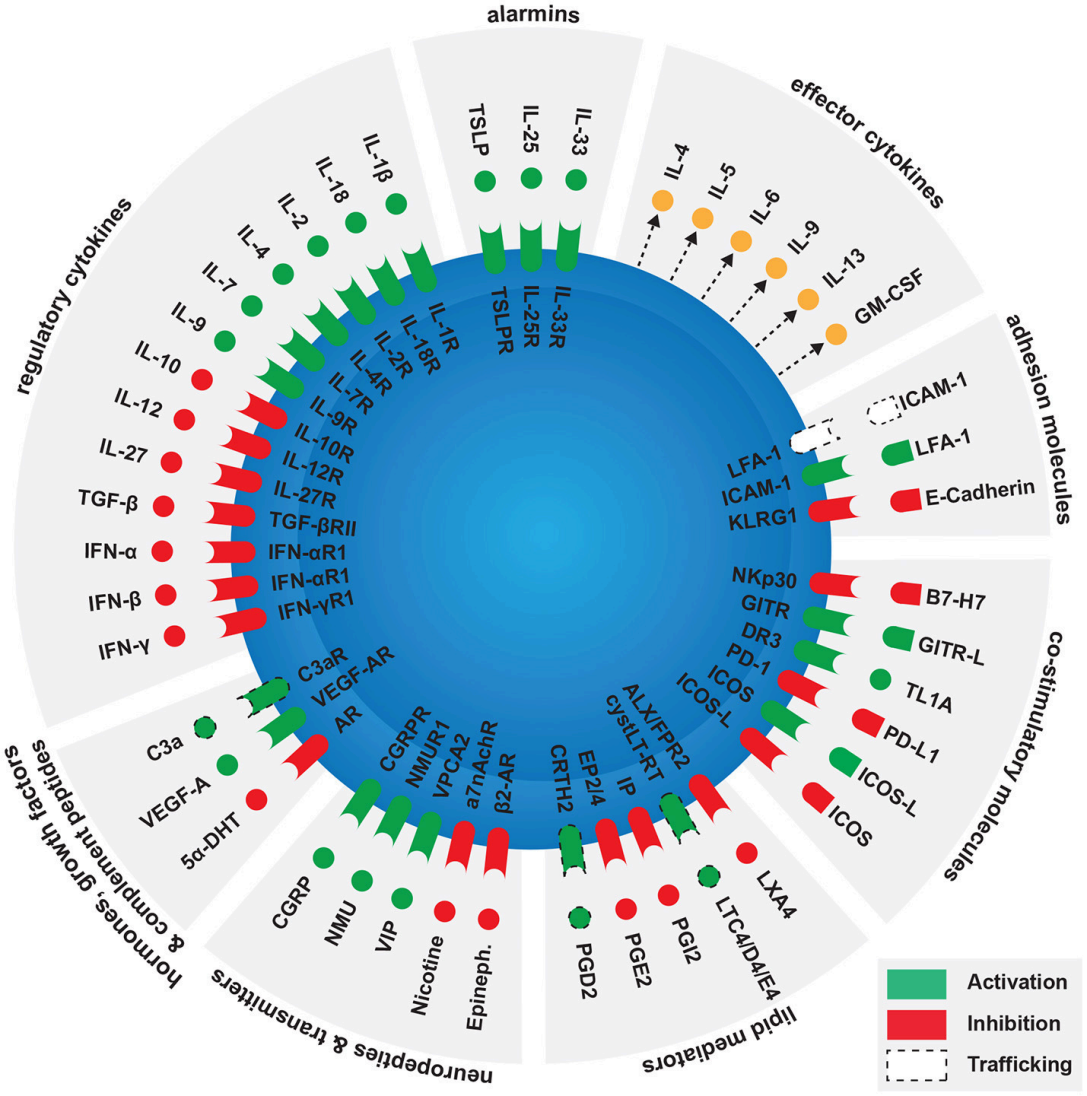
Factors modulating ILC2 activation in the context of allergy. Main ILC2 activators are alarmins released by epithelial cells, as activated ILC2s release various effector cytokines including IL-4, IL-5, IL-6, IL-9, IL-13, and GM-CSF. Several groups of molecules can enhance or inhibit ILC2 activation. These include contact independent pathways such as regulatory cytokines, hormones, growth factors, complement peptides, neuropeptides, neurotransmitters, and lipid mediators. Contact-dependent pathways include co-stimulatory and adhesion molecules.

According to a recent genetic cluster analysis, there are at least five different clinical phenotypes of asthma (25). One cluster showed higher blood and sputum eosinophils, driven by a Th2-dominant inflammatory response (26). Multiple studies have shown that ILC2s are directly involved in eosinophilic asthma (11). They are located near the basement membrane subjacent to the airway epithelium, residing within 70 μm of airway branchpoints (27). This strategic location allows them to act as sentinels and rapidly respond to allergen exposure. The main activators of ILC2s are alarmins released by activated epithelial cells such as **IL-33**, **IL-25**, or **TSLP** (18, 28–32). Activated ILC2s release various effector cytokines including IL-4, IL-5, IL-9, and IL-13 (6–8). IL-5 and IL-13 cause eosinophilia and smooth muscle cell contraction respectively, altogether contributing to the pathogenesis of asthma (33, 34). Interestingly, activated ILC2s also release considerable amounts of pleiotropic cytokines IL-6 and GM-CSF (35–37). While IL-6 is known to induce the development of Th17 cells from naïve T-cells (38), ILC3-derived GM-CSF is a key regulator of oral tolerance to dietary antigens by modulating macrophage effector functions (39). It remains to be elucidated whether ILC2-derived IL-6 and GM-CSF can have similar immunoregulatory effects in the context of allergic diseases. Furthermore, ILC2s contribute to tissue homeostasis through the secretion of amphiregulin (40). Several studies show that activated ILC2s enhance Th2-cell activation in response to allergens (41–43). However, in the absence of T-cells, effector cytokines released by ILC2s in response to intranasal challenge with alarmins are strikingly sufficient to induce airway inflammation and AHR (44, 45). ILC2s are therefore emerging as important players in the pathogenesis of allergic diseases such as asthma, and a better understanding of their function will open the door to the development of new therapeutic strategies. Factors modulating ILC2 functions in allergic diseases will be discussed in this review, summarized in Figure 1.

## Modulation of ILC2 activation

### Regulatory cytokines

We and others recently reported that regulatory T-cells (Treg)-derived cytokines suppress the development of ILC2-dependent lung inflammation (46). Induced Tregs (iTregs), rather than natural Tregs (nTregs) efficiently suppress the production of ILC2-derived IL-5 and IL-13, ultimately inhibiting the development of airway inflammation and AHR. The suppressive effects of Tregs depend on ICOS:ICOSL interactions, but also on the release by Tregs of **IL-10** and **TGF-**β. In line with our results, studies have confirmed the suppressive effects of IL-10 and TGF-β on mouse and human ILC2s in the context of asthma (47) and upper airway inflammation (48). Further studies are however required to better characterize the role of TGF-β, as epithelial-derived TGF-β was recently shown to drive pulmonary inflammation (49).

Interferons have various immunomodulatory functions and are classified in two families: Type I Interferons such as IFN-α and IFN-β, and Type II Interferons such as IFN-γ (50). We recently reported that plasmacytoid DC (pDC)-derived **IFN-**α directly suppresses ILC2 activation in models of asthma (51). In our study, we showed that IFN-α inhibits pulmonary ILC2-derived secretion of IL-5 and IL-13, ultimately preventing the development of airway inflammation and AHR. Depletion of pDCs strikingly reverses the suppressive effects on ILC2s. Interestingly **IFN-**β and **IFN-**γ also strongly suppress pulmonary ILC2 proliferation and cytokine production, suggesting that both Type I and Type II Interferons can dampen ILC2-derived lung inflammation (52, 53).

The classical view of the Th1/Th2 paradigm supports that Th1 cytokines inhibit Th2 cell differentiation and *vice versa* (54). This holds true for ILC2s, as they resemble Th2 cells. **IL-12**, a Th1 cytokine, reduces ILC2 activation and promotes their transition to T-bet^high^ GATA-3^low^ ILCs (55), suggesting that ILC2s retain plasticity. Furthermore, **IL-27**, a member of the IL-12 cytokine family, suppresses ILC2 cytokine production in the lungs (53, 56). On the other hand, Th2 cytokines were reported to enhance ILC2 cytokine production. A study shows in a model of lung inflammation that key type 2 cytokine **IL-4** derived from basophils enhances ILC2 secretion of IL-5 and IL-13, ultimately favoring eosinophilia (57). Besides being known as a Th9 cell signature cytokine, **IL-9** is also required for the survival and homeostasis of ILC2s (58, 59). Furthermore, lung ILC2s themselves secrete IL-9 as autocrine IL-9 is crucial for ILC2 effector functions (60, 61). Similar to T-cells, ILC2s require survival factors for efficient activation (62). **IL-2** is crucial in the maintenance of ILC2 activation (43, 63), as they further rely on **IL-7** for their development (64, 65) and efficient activation (20). A recent report however reveals that IL-7 is not strictly required for the development of ILC2s (66). Interestingly, human lung ILC2s are further activated by **IL-1**β and closely related cytokine **IL-18** (37, 67). In addition to affecting ILC2 activation, several lines of evidence suggest that cytokines from the local microenvironment affect ILC2 plasticity depending on the context. ILC2s may develop into specific subsets or even express an ILC1-phenotype, although further studies are warranted to better understand such processes (68–70).

### Co-stimulatory molecules

ILC2s express multiple receptors on their surface that bind to ligands present on other immune cells. Among those are co-stimulatory molecules, known to modulate T-cell activation (71). Both mouse and human lung ILC2s express Inducible T-cell co-stimulator (ICOS) at steady state but also upon inflammation (72–75), and we were the first to show that they also express ligand **ICOS-L** (76). We show that the ICOS:ICOS-L *trans*-interaction is crucial for ILC2 homeostasis and effector functions in models of lung inflammation. Compared to controls, mice genetically deficient in ICOS develop less AHR and lung inflammation as a result of a defect in pulmonary ILC2-derived IL-5 and IL-13 secretion and increased apoptotic rates. The observed effects were strictly ILC2-dependent, as alymphoid mice adoptively transferred with ICOS-deficient ILC2s develop less AHR compared to control mice. In a separate study, we further studied the interaction of pulmonary ILC2 surface expression of ICOS with its ligand on T-cells. Strikingly, we found that binding of iTregs via ICOS-L to ILC2s via **ICOS** inhibits ILC2 cytokine secretion and development of AHR (46). A recent study reveals that **PD-1**, another co-stimulatory molecule, is expressed on ILC2s and acts as a negative regulator of ILC2s by inhibiting proliferation and IL-13 cytokine production in a model of lung inflammation (77). Although PD-1 is expressed on ILC2s, further studies are required to better understand its function in the context of allergic asthma. For example, it is not clear whether a PD-1 agonist can be used as a therapeutic agent in the context of ILC2-dependent asthma.

Members of the tumor necrosis factor receptor superfamily (TNFRSF) and their ligands (TNFSF) provide key co-stimulatory signals to T-cells (78). They contribute to T-cell homeostasis and induce or restrict immune responses. Such findings have led to the design of treatments of autoimmune diseases and tumors (79, 80). Similar to T-cells, several TNFRSF and their ligands are involved in ILC2 homeostasis and activation in the context of allergic diseases. **TNFRSF25** (DR3) was first shown to be required for mouse and human ILC2 expansion and function (81, 82). ILC2s express TNFRSF25, and engagement with TNFSF15 induces ILC2 expansion, survival and cytokine secretion in the lungs (82). Interestingly, TNFSF15 alone is sufficient to activate ILC2s. In a mouse model of dermatitis, a recent study further shows that TNFSF15 activates skin ILC2s in a TNFRSF25-dependent manner (83). A report describes the co-stimulatory role of **TNFRSF18** (GITR) in ILC2-dependent lung inflammation (61). Interaction of the receptor with GITR-L (DTA-1) enhances autocrine IL-9-induced IL-5 and IL-13 secretion by ILC2s, ultimately driving lung inflammation. However, further studies are required to better characterize the function of TNFRSF18 on ILC2s during inflammation. Upregulation of the tumor-associated surface molecule B7-H7 is observed in human AD lesions (84). Binding to its receptor **NKp30** on ILC2s induces type 2 cytokine secretion, suggesting this pathway may be involved in ILC2-derived skin inflammation (84). Although no effect on ILC2 activation was reported, a recent study describes ILC2s as a source of TNFSF4 (OX40L), promoting IL-33-driven Th2 and Treg lung inflammation by binding to TNFRSF4 (OX40) (85, 86). Altogether, these studies suggest that co-stimulatory molecules are potent modulators of ILC2 activation.

### Lipid mediators

Lipids are generally known as a source of energy for the human body and crucial components of cellular membranes (87). However, eicosanoids such as prostaglandins (PG), cysteinyl leukotrienes (cystLT) and lipoxins (LX) are bioactive lipids also involved in cell signaling (88, 89). In the context of ILC2-driven allergic inflammation, several prostaglandins were described to modulate ILC2 functions (90–92). The most studied prostaglandin is **PGD2**, which binds to CRTH2 and induces human and mouse ILC2 chemotaxis and type 2 cytokine production in the inflamed lungs (90, 93, 94). Unlike PGD2, other prostaglandins were shown to inhibit ILC2 functions (91, 92). In a mouse model of airway inflammation, **PGI2** binds to PGI2 receptor IP on ILC2s and reduces the number of lung-expressing IL-5 and IL-13 ILC2s (91). Another recent study shows that **PGE2** inhibits human tonsillar ILC2 proliferation and cytokine secretion by binding to EP2 and EP4 on ILC2s (92). Cysteinyl leukotrienes are important inflammatory mediators in the context of allergy. Mouse and human ILC2s express cystLT-R1, as **LTD4** was first shown to induce ILC2 proliferation and production of cytokines during lung inflammation (95). Furthermore, it was recently reported that **LTC4** given intranasally with low dose IL-33 increases lung ILC2 proliferation and type 2 cytokine secretion in mice (96). In another recent study using an AD model, **LTE4** induces migration, reduces apoptosis and enhances cytokine secretion in human ILC2s (97). Finally, lipoxins are are generally associated with resolution of inflammation (98), with **LXA4** shown to inhibit IL-13 production on activated human ILC2s (99). Altogether, these studies suggest that lipid mediators are potent modulators of ILC2 activation and chemotaxis.

### Adhesion molecules

Besides their role in cell migration, adhesion molecules are required for efficient, tight cell-to-cell interactions and can function as co-stimulatory molecules (100). ILC2s express integrins such as leukocyte function-associated molecule (LFA-1, α_L_β_2_), interacting with members of the intercellular cell adhesion molecule (ICAM) family of ligands (101). Interestingly, disruption of LFA-1 and ICAM-1 binding impaired the development of airway inflammation (102). A study recently showed that mouse and human ILC2s express both LFA-1 and ICAM-1 (103). This study elegantly shows that **LFA-1** is required for ILC2 migration from the circulation to the lungs during airway inflammation, although it did not affect ILC2 functions. This study strongly supports that ILC2s are not only resident cells and can also be recruited to inflamed lungs, an observation also made by others (104). Besides these observed effects, **ICAM-1** is furthermore required for ILC2 homeostasis and efficient activation in the lungs, as absence of ICAM-1 specifically on ILC2s significantly inhibits IL-5 and IL-13 secretion and development of airway inflammation (105). **Killer cell lectin-like receptor G1 (KLRG1)** - E-cadherin interactions inhibit ILC2 activation in the context of human AD lesions (19). Skin ILC2s of AD lesions express high levels of KLRG1 compared to healthy controls. Interaction with E-cadherin, an adhesion molecule found on epithelial cells, reduces ILC2 proliferation and cytokine secretion in response to both IL-25 and IL-33. The KLRG1-E-cadherin interaction is of particular interest, as loss of E-cadherin on lung epithelium in humans is linked to asthma severity (106). Altogether, adhesion molecules are emerging as efficient modulators of ILC2 homeostasis, function, and trafficking.

### Neuropeptides and neurotransmitters

Neuropeptides and neurotransmitters are proteins used by neurons to communicate with each other. Interestingly, the immune and nervous systems are closely linked (107). Neurotransmitter acetylcholine binds to receptors including nicotinic acetylcholine receptors (nAChRs). As opposed to muscarinic AChRs, nAChRs also respond to nicotine, ultimately affecting immune responses (108). We were the first to show that human and mouse ILC2s express **a7nAChR**, regulating ILC2-mediated lower airway inflammation and AHR (109). Engagement of the a7nAChR with GTS-21, a specific agonist, inhibits ILC2-derived IL-5 and IL-13 secretion, ultimately inhibiting the development of airway inflammation and AHR. ILC2s also express β**2-adrenergic receptor (**β**2-AR)**, known to interact with neurotransmitter epinephrine, and was recently described as a negative modulator of ILC2 activation. This study shows that mice treated with a β2-AR agonist exhibit less lung ILC2 proliferation and cytokine production in response to IL-33 (110).

Neuropeptides can amplify allergic lung inflammation (27, 111). First, a study shows that ILC2s express Neuromedin U receptor 1 (NMUR1) at steady state and during inflammation (111). NMUR1 ligand **Neuromedin U (NMU)** amplifies IL-25-induced allergic lung inflammation by increasing ILC2-derived IL-5 and IL-13 secretion. As a result, mice co-challenged with NMU and IL-25 develop increased airway inflammation and AHR compared to controls. Another study recently shows that neuropeptide **calcitonin gene-related peptide (CGRP)** induces ILC2-driven allergic lung inflammation by enhancing ILC2-derived IL-5 secretion (27). Interestingly, the source of CGRP are pulmonary neuroendocrine cells (PNECs) that reside in close proximity to ILC2s at airway branchpoints. Additionally, lung ILC2s release IL-5 after stimulation with the neuropeptide **vasoactive intestinal peptide (VIP)**. VIP signals through VIP receptor type 2 (VPCA2) expressed on ILC2s, as they release IL-5 after stimulation with a specific agonist (34). Altogether, these studies suggest that neuronal products are emerging as potent modulators of ILC2 activation.

### Hormones, growth factors and complement peptides

Studies have shown that asthma incidence differs depending on the sex (112). In human asthmatics, the number of blood ILC2s is increased in women compared to men (113). This highly suggests a role for sex hormones as regulators of the development of asthma, as they are already known to affect T-cell differentiation and cytokine secretion in a different context (114, 115). Three studies recently established a role for androgens in ILC2-driven airway inflammation (113, 116, 117). A study shows that male mice develop less severe IL-33-induced allergic asthma compared to females (116). Interestingly, this difference is due to an increase in **androgen receptor (AR)** signaling, which ultimately decreases ILC2-dependent airway inflammation. In line with this, a group recently found that a derivative of testosterone, **5**α**-dihydrotestosterone (5**α**-DHT)** directly inhibits lung ILC2 proliferation and secretion of IL-5 and IL-13 in response to IL-33 (113). As a result, testosterone decreases Alternaria extract-induced airway inflammation. Interestingly, lung ILC2s from gonadectomized females secrete less IL-5 compared to controls, suggesting that ovarian hormones may also affect ILC2 homeostasis and function (118). In line with this, ILC2s were shown to be regulated by female sex hormones in the uterus (119).

Members of the vascular endothelial growth factors (VEGF) including VEGF-A, VEGF-C, and VEGF-D are secreted by multiple immune cells (120). Recently, a study shows that both human and mouse ILC2s strikingly promote AHR via the production of **VEGF-A** (121). ILC2s stimulated with IL-33 release autocrine VEGF-A, which binds to surface VEGFR2, altogether promoting cytokine secretion and lung inflammation. Treatment of mice with a specific VEGFR2 inhibitor significantly inhibits the development of AHR in response to allergen. Interestingly, ILC2s from patients with asthma express increased VEGF-A transcripts.

Finally, complement system activation and generation of anaphylatoxins, or complement peptides, induces and regulates the development of type 2 responses at mucosal surfaces (122). A study recently shows that **complement peptide C3a** increases ILC2 numbers in the lungs, as well as their secretion of IL-13 and GM-CSF in response to IL-33 (36). This novel mechanism by which C3a drives type 2 immunity in the lungs is of particular interest as elevated levels of C3a were found in asthmatics airways (123).

### Concluding remarks and future directions

ILC2s are potent producers of type 2 cytokines, and it is therefore not surprising that they are involved in the development of various allergic diseases including asthma. Treating such diseases by targeting upstream ILC2 activators such as alarmins will likely have unwanted adverse effects on other immunological pathways. In recent years however, multiple pathways were described to modulate ILC2 effector functions, ultimately affecting the pathogenesis of allergic diseases. Such findings provide valuable information for the design of novel therapeutic strategies, largely dependent on corticosteroids in the context of asthma and AD. Open questions however remain to be tackled. First, although research over the past years has revealed the expression of multiple key receptors on ILC2s, no specific marker has yet to be identified (Table 1). Second, although ILC2s are considered as resident cells (124), emerging data suggests that they express chemotactic/trafficking molecules upon inflammation and are therefore also recruited to inflamed tissues, as discussed (103, 104). A better understanding of ILC2 trafficking and tissue tropism will provide valuable information for the treatment of allergic diseases. Third, several lines of evidence suggest that ILC2s retain plasticity and adapt to signals from the local microenvironment, such as composition of the local cytokine pool. ILC2s can develop into specific sub phenotypes, such as the newly described IL-10 producing ILC2_10_ (69) or IL-17 producing ILC2_17_ (70). Furthermore, several lines of evidence describe conversion from ILC2 to an ILC1-like phenotype such as following smoke exposure (68). It will be crucial to delineate the factors inducing plasticity or commitment among ILC2s particularly in the context of allergic diseases. Finally, since asthma is a heterogeneous disease, it will be essential to find a biomarker characterizing the cohort of asthma patients with increased airway ILC2 activity.

## Author contributions

BH wrote the manuscript and designed the Figure. PS contributed to writing the manuscript and Figure design. OA supervised and edited the manuscript.

### Conflict of interest statement

The authors declare that the research was conducted in the absence of any commercial or financial relationships that could be construed as a potential conflict of interest.

## References

[1] Pulendran B Artis D. New paradigms in type 2 immunity. Science (2012) 337:431–5. 10.1126/science.1221064 22837519 PMC4078898

[2] Shimbara A Christodoulopoulos P Soussi-Gounni A Olivenstein R Nakamura Y Levitt RC. IL-9 and its receptor in allergic and nonallergic lung disease: increased expression in asthma. J Allergy Clin Immunol. (2000) 105:108–15. 10.1016/S0091-6749(00)90185-4 10629460

[3] King C Tangye SG Mackay CR. T follicular helper (TFH) cells in normal and dysregulated immune responses. Annu Rev Immunol. (2008) 26:741–66. 10.1146/annurev.immunol.26.021607.090344 18173374

[4] Liang HE Reinhardt RL Bando JK Sullivan BM Ho IC Locksley RM. Divergent expression patterns of IL-4 and IL-13 define unique functions in allergic immunity. Nat Immunol. (2011) 13:58–66. 10.1038/ni.2182 22138715 PMC3242938

[5] Akbari O Stock P Meyer E Kronenberg M Sidobre S Nakayama T. Essential role of NKT cells producing IL-4 and IL-13 in the development of allergen-induced airway hyperreactivity. Nat Med. (2003) 9:582–8. 10.1038/nm851 12669034

[6] Moro K Yamada T Tanabe M Takeuchi T Ikawa T Kawamoto H. Innate production of T(H)2 cytokines by adipose tissue-associated c-Kit(+)Sca-1(+) lymphoid cells. Nature (2010) 463:540–4. 10.1038/nature08636 20023630

[7] Neill DR Wong SH Bellosi A Flynn RJ Daly M Langford TK. Nuocytes represent a new innate effector leukocyte that mediates type-2 immunity. Nature (2010) 464:1367–70. 10.1038/nature08900 20200518 PMC2862165

[8] Price AE Liang HE Sullivan BM Reinhardt RL Eisley CJ Erle DJ. Systemically dispersed innate IL-13-expressing cells in type 2 immunity. Proc Natl Acad Sci USA. (2010) 107:11489–94. 10.1073/pnas.1003988107 20534524 PMC2895098

[9] Vivier E Artis D Colonna M Diefenbach A Di Santo JP Eberl G. Innate lymphoid cells: 10 Years On. Cell (2018) 174:1054–66. 10.1016/j.cell.2018.07.017 30142344

[10] Licona-Limon P Kim LK Palm NW Flavell RA. TH2, allergy and group 2 innate lymphoid cells. Nat Immunol. (2013) 14:536–42. 10.1038/ni.2617 23685824

[11] Maazi H Akbari O. Type two innate lymphoid cells: the Janus cells in health and disease. Immunol Rev. (2017) 278:192–206. 10.1111/imr.12554 28658553 PMC5492968

[12] Mjosberg JM Trifari S Crellin NK Peters CP van Drunen CM Piet B. Human IL-25- and IL-33-responsive type 2 innate lymphoid cells are defined by expression of CRTH2 and CD161. Nat Immunol. (2011) 12:1055–62. 10.1038/ni.2104 21909091

[13] Shaw JL Fakhri S Citardi MJ Porter PC Corry DB Kheradmand F. IL-33-responsive innate lymphoid cells are an important source of IL-13 in chronic rhinosinusitis with nasal polyps. Am J Respir Crit Care Med. (2013) 188:432–9. 10.1164/rccm.201212-2227OC 23805875 PMC5448506

[14] Miljkovic D Bassiouni A Cooksley C Ou J Hauben E Wormald PJ. Association between group 2 innate lymphoid cells enrichment, nasal polyps and allergy in chronic rhinosinusitis. Allergy (2014) 69:1154–61. 10.1111/all.12440 24924975

[15] Walford HH Lund SJ Baum RE White AA Bergeron CM Husseman J. Increased ILC2s in the eosinophilic nasal polyp endotype are associated with corticosteroid responsiveness. Clin Immunol. (2014) 155:126–35. 10.1016/j.clim.2014.09.007 25236785 PMC4254351

[16] Ho J Bailey M Zaunders J Mrad N Sacks R Sewell W Group 2 innate lymphoid cells (ILC2s) are increased in chronic rhinosinusitis with nasal polyps or eosinophilia. Clin Exp Allergy (2015) 45:394–403. 10.1111/cea.12462 25429730

[17] Poposki JA Klingler AI Tan BK Soroosh P Banie H Lewis G. Group 2 innate lymphoid cells are elevated and activated in chronic rhinosinusitis with nasal polyps. Immun Inflamm Dis. (2017) 5:233–43. 10.1002/iid3.161 28474861 PMC5569375

[18] Kim BS Siracusa MC Saenz SA Noti M Monticelli LA Sonnenberg GF. TSLP elicits IL-33-independent innate lymphoid cell responses to promote skin inflammation. Sci Transl Med. (2013) 5:170ra116. 10.1126/scitranslmed.3005374 23363980 PMC3637661

[19] Salimi M Barlow JL Saunders SP Xue L Gutowska-Owsiak D Wang X. A role for IL-25 and IL-33-driven type-2 innate lymphoid cells in atopic dermatitis. J Exp Med. (2013) 210:2939–50. 10.1084/jem.20130351 24323357 PMC3865470

[20] Bartemes KR Kephart GM Fox SJ Kita H. Enhanced innate type 2 immune response in peripheral blood from patients with asthma. J Allergy Clin Immunol. (2014) 134:671–8.e674. 10.1016/j.jaci.2014.06.024 25171868 PMC4149890

[21] Christianson CA Goplen NP Zafar I Irvin C Good JT Jr Rollins DR. Persistence of asthma requires multiple feedback circuits involving type 2 innate lymphoid cells and IL-33. J Allergy Clin Immunol. (2015) 136:59–68.e14. 10.1016/j.jaci.2014.11.037 25617223 PMC4494983

[22] Liu T Wu J Zhao J Wang J Zhang Y Liu L. Type 2 innate lymphoid cells: a novel biomarker of eosinophilic airway inflammation in patients with mild to moderate asthma. Respir Med. (2015) 109:1391–6. 10.1016/j.rmed.2015.09.016 26459159

[23] Nagakumar P Denney L Fleming L Bush A Lloyd CM Saglani S. Type 2 innate lymphoid cells in induced sputum from children with severe asthma. J Allergy Clin Immunol. (2016) 137:624–6.e626. 10.1016/j.jaci.2015.06.038 26277593

[24] Smith SG Chen R Kjarsgaard M Huang C Oliveria JP O'Byrne PM. Increased numbers of activated group 2 innate lymphoid cells in the airways of patients with severe asthma and persistent airway eosinophilia. J Allergy Clin Immunol. (2016) 137:75–86.e78. 10.1016/j.jaci.2015.05.037 26194544

[25] Moore WC Meyers DA Wenzel SE Teague WG Li H Li X. Identification of asthma phenotypes using cluster analysis in the severe asthma research program. Am J Respir Crit Care Med. (2010) 181:315–23. 10.1164/rccm.200906-0896OC 19892860 PMC2822971

[26] Woodruff PG Modrek B Choy DF Jia G Abbas AR Ellwanger A. T-helper Type 2–driven inflammation defines major subphenotypes of asthma. Am J Respir Crit Care Med. (2009) 180:388–95. 10.1164/rccm.200903-0392OC 19483109 PMC2742757

[27] Sui P Wiesner DL Xu J Zhang Y Lee J Van Dyken S. Pulmonary neuroendocrine cells amplify allergic asthma responses. Science (2018) 360:eaan8546. 10.1126/science.aan8546 29599193 PMC6387886

[28] Al-Shami A. A role for TSLP in the development of inflammation in an asthma model. J Exp Med. (2005) 202:829–39. 10.1084/jem.20050199 16172260 PMC2212950

[29] Pichery M Mirey E Mercier P Lefrancais E Dujardin A Ortega N. Endogenous IL-33 is highly expressed in mouse epithelial barrier tissues, lymphoid organs, brain, embryos, and inflamed tissues: *in situ* analysis using a novel Il-33-LacZ gene trap reporter strain. J Immunol. (2012) 188:3488–95. 10.4049/jimmunol.1101977 22371395

[30] Barlow JL Peel S Fox J Panova V Hardman CS Camelo A. IL-33 is more potent than IL-25 in provoking IL-13-producing nuocytes (type 2 innate lymphoid cells) and airway contraction. J Allergy Clin Immunol. (2013) 132:933–41. 10.1016/j.jaci.2013.05.012 23810766

[31] Iijima K Kobayashi T Hara K Kephart GM Ziegler SF McKenzie AN. IL-33 and thymic stromal lymphopoietin mediate immune pathology in response to chronic airborne allergen exposure. J Immunol. (2014) 193:1549–59. 10.4049/jimmunol.1302984 25015831 PMC4119518

[32] Anderson EL Kobayashi T Iijima K Bartemes KR Chen CC Kita H. IL-33 mediates reactive eosinophilopoiesis in response to airborne allergen exposure. Allergy (2016) 71:977–88. 10.1111/all.12861 26864308 PMC5107318

[33] Tliba O Deshpande D Chen H Van Besien C Kannan M Panettieri RA. IL-13 enhances agonist-evoked calcium signals and contractile responses in airway smooth muscle. Br J Pharmacol. (2003) 140:1159–62. 10.1038/sj.bjp.0705558 14597600 PMC1574143

[34] Nussbaum JC Van Dyken SJ von Moltke J Cheng LE Mohapatra A Molofsky AB. Type 2 innate lymphoid cells control eosinophil homeostasis. Nature (2013) 502:245–8. 10.1038/nature12526 24037376 PMC3795960

[35] Mjosberg J Bernink J Golebski K Karrich JJ Peters CP Blom B. The transcription factor GATA3 is essential for the function of human type 2 innate lymphoid cells. Immunity (2012) 37:649–59. 10.1016/j.immuni.2012.08.015 23063330

[36] Gour N Smole U Yong HM Lewkowich IP Yao N Singh A. C3a is required for ILC2 function in allergic airway inflammation. Mucosal Immunol. (2018). [Epub ahead of print]. 10.1038/s41385-018-0064-x 30104625 PMC6279480

[37] Simoni Y Fehlings M Kloverpris HN McGovern N Koo SL Loh CY. Human innate lymphoid cell subsets possess tissue-type based heterogeneity in phenotype and frequency. Immunity (2018) 48:1060. 10.1016/j.immuni.2018.04.028 29768165

[38] Kimura A Kishimoto T. IL-6: regulator of Treg/Th17 balance. Eur J Immunol. (2010) 40:1830–5. 10.1002/eji.201040391 20583029

[39] Mortha A Chudnovskiy A Hashimoto D Bogunovic M Spencer SP Belkaid Y. Microbiota-dependent crosstalk between macrophages and ILC3 promotes intestinal homeostasis. Science (2014) 343:1249288. 10.1126/science.1249288 24625929 PMC4291125

[40] Monticelli LA Sonnenberg GF Abt MC Alenghat T Ziegler CGK Doering TA Innate lymphoid cells promote lung tissue homeostasis following acute influenza virus infection. Nat Immunol. (2011) 12:1045–54. 10.1031/ni.2131 21946417 PMC3320042

[41] Halim TY Steer CA Matha L Gold MJ Martinez-Gonzalez I McNagny KM. Group 2 innate lymphoid cells are critical for the initiation of adaptive T helper 2 cell-mediated allergic lung inflammation. Immunity (2014) 40:425–35. 10.1016/j.immuni.2014.01.011 24613091 PMC4210641

[42] Mirchandani AS Besnard AG Yip E Scott C Bain CC Cerovic V. Type 2 innate lymphoid cells drive CD4+ Th2 cell responses. J Immunol. (2014) 192:2442–8. 10.4049/jimmunol.1300974 24470502

[43] Oliphant CJ Hwang YY Walker JA Salimi M Wong SH Brewer JM. MHCII-mediated dialog between group 2 innate lymphoid cells and CD4(+) T cells potentiates type 2 immunity and promotes parasitic helminth expulsion. Immunity (2014) 41:283–95. 10.1016/j.immuni.2014.06.016 25088770 PMC4148706

[44] Bartemes KR Iijima K Kobayashi T Kephart GM McKenzie AN Kita H. IL-33-responsive lineage- CD25+ CD44(hi) lymphoid cells mediate innate type 2 immunity and allergic inflammation in the lungs. J Immunol. (2012) 188:1503–13. 10.4049/jimmunol.1102832 22198948 PMC3262877

[45] Kim HY Chang YJ Subramanian S Lee HH Albacker LA Matangkasombut P. Innate lymphoid cells responding to IL-33 mediate airway hyperreactivity independently of adaptive immunity. J Allergy Clin Immunol. (2012) 129:216–27.e6. 10.1016/j.jaci.2011.10.036 22119406 PMC3246069

[46] Rigas D Lewis G Aron JL Wang B Banie H Sankaranarayanan I. Type 2 innate lymphoid cell suppression by regulatory T cells attenuates airway hyperreactivity and requires inducible T-cell costimulator-inducible T-cell costimulator ligand interaction. J Allergy Clin Immunol. (2017) 139:1468–77.e2. 10.1016/j.jaci.2016.08.034 27717665 PMC5378695

[47] Krishnamoorthy N Burkett PR Dalli J Abdulnour REE Colas R Ramon S. Maresin-1 engages regulatory T cells to limit type 2 innate lymphoid cell activation and promote resolution of lung inflammation. J Immunol. (2015) 194:863–7. 10.4049/jimmunol.1402534 25539814 PMC4297713

[48] Ogasawara N Poposki JA Klingler AI Tan BK Weibman AR Hulse KE. IL-10, TGF-beta, and glucocorticoid prevent the production of type 2 cytokines in human group 2 innate lymphoid cells. J Allergy Clin Immunol. (2018) 141:1147–51.e1148. 10.1016/j.jaci.2017.09.025 29074458 PMC5844803

[49] Denney L Byrne AJ Shea TJ Buckley JS Pease JE Herledan GMF. Pulmonary epithelial cell-derived cytokine TGF-β1 is a critical cofactor for enhanced innate lymphoid cell function. Immunity (2015) 43:945–58. 10.1016/j.immuni.2015.10.012 26588780 PMC4658339

[50] Platanias LC. Mechanisms of type-I- and type-II-interferon-mediated signalling. Nat Rev Immunol. (2005) 5:375–86. 10.1038/nri1604 15864272

[51] Maazi H Banie H Aleman Muench GR Patel N Wang B Sankaranarayanan I. Activated plasmacytoid dendritic cells regulate type 2 innate lymphoid cell-mediated airway hyperreactivity. J Allergy Clin Immunol. (2018) 141:893–905.e6. 10.1016/j.jaci.2017.04.043 28579374

[52] Duerr CU McCarthy CD Mindt BC Rubio M Meli AP Pothlichet J. Type I interferon restricts type 2 immunopathology through the regulation of group 2 innate lymphoid cells. Nat Immunol. (2016) 17:65–75. 10.1038/ni.3308 26595887 PMC9135352

[53] Moro K Kabata H Tanabe M Koga S Takeno N Mochizuki M. Interferon and IL-27 antagonize the function of group 2 innate lymphoid cells and type 2 innate immune responses. Nat Immunol. (2016) 17:76–86. 10.1038/ni.3309 26595888

[54] Romagnani S. The Th1/Th2 paradigm. Immunol Today (1997) 18:263–6. 9190109 10.1016/s0167-5699(97)80019-9

[55] Almeida FF Belz GT. Innate lymphoid cells: models of plasticity for immune homeostasis and rapid responsiveness in protection. Mucosal Immunol. (2016) 9:1103–12. 10.1038/mi.2016.64 27484190

[56] McHedlidze T Kindermann M Neves AT Voehringer D Neurath MF Wirtz S. IL-27 suppresses type 2 immune responses *in vivo* via direct effects on group 2 innate lymphoid cells. Mucosal Immunol. (2016) 9:1384–94. 10.1038/mi.2016.20 26982595

[57] Motomura Y Morita H Moro K Nakae S Artis D Endo TA. Basophil-derived interleukin-4 controls the function of natural helper cells, a member of ILC2s, in lung inflammation. Immunity (2014) 40:758–71. 10.1016/j.immuni.2014.04.013 24837103

[58] Wilhelm C Hirota K Stieglitz B Van Snick J Tolaini M Lahl K. An IL-9 fate reporter demonstrates the induction of an innate IL-9 response in lung inflammation. Nat Immunol. (2011) 12:1071–7. 10.1038/ni.2133 21983833 PMC3198843

[59] Turner JE Morrison PJ Wilhelm C Wilson M Ahlfors H Renauld JC. IL-9-mediated survival of type 2 innate lymphoid cells promotes damage control in helminth-induced lung inflammation. J Exp Med. (2013) 210:2951–65. 10.1084/jem.20130071 24249111 PMC3865473

[60] Mohapatra A Van Dyken SJ Schneider C Nussbaum JC Liang H Locksley RM. Group 2 innate lymphoid cells utilize the IRF4-IL-9 module to coordinate epithelial cell maintenance of lung homeostasis. Mucosal Immunol. (2016) 9:275–86. 10.1038/mi.2015.59 26129648 PMC4698110

[61] Nagashima H Okuyama Y Fujita T Takeda T Motomura Y Moro K. GITR cosignal in ILC2s controls allergic lung inflammation. J Allergy Clin Immunol. (2018) 141:1939–43.e8. 10.1016/j.jaci.2018.01.028 29427641

[62] Kelly E Won A Refaeli Y Van Parijs L. IL-2 and related cytokines can promote T cell survival by activating AKT. J Immunol. (2002) 168:597–603. 10.4049/jimmunol.168.2.597 11777951

[63] Roediger B Kyle R Tay SS Mitchell AJ Bolton HA Guy TV. IL-2 is a critical regulator of group 2 innate lymphoid cell function during pulmonary inflammation. J Allergy Clin Immunol. (2015) 136:1653–63.e1657. 10.1016/j.jaci.2015.03.043 26025126

[64] Vonarbourg C Diefenbach A. Multifaceted roles of interleukin-7 signaling for the development and function of innate lymphoid cells. Semin Immunol. (2012) 24:165–74. 10.1016/j.smim.2012.03.002 22541512

[65] Diefenbach A Colonna M Koyasu S. Development, differentiation, and diversity of innate lymphoid cells. Immunity (2014) 41:354–65. 10.1016/j.immuni.2014.09.005 25238093 PMC4171710

[66] Robinette ML Bando JK Song W Ulland TK Gilfillan S Colonna M. IL-15 sustains IL-7R-independent ILC2 and ILC3 development. Nat Commun. (2017) 8:14601. 10.1038/ncomms14601 28361874 PMC5380969

[67] Bal SM Bernink JH Nagasawa M Groot J Shikhagaie MM Golebski K. IL-1beta, IL-4 and IL-12 control the fate of group 2 innate lymphoid cells in human airway inflammation in the lungs. Nat Immunol. (2016) 17:636–45. 10.1038/ni.3444 27111145

[68] Silver JS Kearley J Copenhaver AM Sanden C Mori M Yu L Inflammatory triggers associated with exacerbations of COPD orchestrate plasticity of group 2 innate lymphoid cells in the lungs. Nat Immunol. (2016) 17:626–35. 10.1038/ni.3443 27111143 PMC5345745

[69] Seehus CR Kadavallore A Torre B Yeckes AR Wang Y Tang J. Alternative activation generates IL-10 producing type 2 innate lymphoid cells. Nat Commun. (2017) 8:1900. 10.1038/s41467-017-02023-z 29196657 PMC5711851

[70] Cai T Qiu J Ji Y Li W Ding Z Suo C. IL-17-producing ST2(+) group 2 innate lymphoid cells play a pathogenic role in lung inflammation. J Allergy Clin Immunol. (2018). [Epub ahead of print]. 10.1016/j.jaci.2018.03.007 29625134 PMC6170730

[71] Chen L. Molecular mechanisms of T cell co-stimulation and co-inhibition. Nat Rev Immunol. (2013) 13:227–42. 10.1038/nri3405 23470321 PMC3786574

[72] Barlow JL Bellosi A Hardman CS Drynan LF Wong SH Cruickshank JP. Innate IL-13-producing nuocytes arise during allergic lung inflammation and contribute to airways hyperreactivity. J Allergy Clin Immunol. (2012) 129:191–8.e191–4. 10.1016/j.jaci.2011.09.041 22079492

[73] Kamachi F Isshiki T Harada N Akiba H Miyake S. ICOS promotes group 2 innate lymphoid cell activation in lungs. Biochem Biophys Res Commun. (2015) 463:739–45. 10.1016/j.bbrc.2015.06.005 26049110

[74] Maazi H Akbari O. ICOS regulates ILC2s in asthma. Oncotarget (2015) 6:24584–5. 10.18632/oncotarget.5245 26309088 PMC4694773

[75] Paclik D Stehle C Lahmann A Hutloff A Romagnani C. ICOS regulates the pool of group 2 innate lymphoid cells under homeostatic and inflammatory conditions in mice. Eur J Immunol. (2015) 45:2766–72. 10.1002/eji.201545635 26249010

[76] Maazi H Patel N Sankaranarayanan I Suzuki Y Rigas D Soroosh P. ICOS:ICOS-ligand interaction is required for type 2 innate lymphoid cell function, homeostasis, and induction of airway hyperreactivity. Immunity (2015) 42:538–51. 10.1016/j.immuni.2015.02.007 25769613 PMC4366271

[77] Taylor S Huang Y Mallett G Stathopoulou C Felizardo TC Sun MA. PD-1 regulates KLRG1(+) group 2 innate lymphoid cells. J Exp Med. (2017) 214:1663–78. 10.1084/jem.20161653 28490441 PMC5461001

[78] Ward-Kavanagh L Lin WW Šedý JS Ware CF. The TNF receptor superfamily in costimulating and coinhibitory responses. Immunity (2016) 44:1005–19. 10.1016/j.immuni.2016.04.019 27192566 PMC4882112

[79] Martinez Gomez JM Croxford JL Yeo KP Angeli V Schwarz H Gasser S. Development of experimental autoimmune encephalomyelitis critically depends on CD137 ligand signaling. J Neurosci. (2012) 32:18246–52. 10.1523/jneurosci.2473-12.2012 23238738 PMC6621746

[80] Redmond WL Linch SN Kasiewicz MJ. Combined targeting of costimulatory (OX40) and coinhibitory (CTLA-4) pathways elicits potent effector T cells capable of driving robust antitumor immunity. Cancer Immunol Res. (2014) 2:142–53. 10.1158/2326-6066.Cir-13-0031-t 24778278 PMC4007342

[81] Meylan F Hawley ET Barron L Barlow JL Penumetcha P Pelletier M. The TNF-family cytokine TL1A promotes allergic immunopathology through group 2 innate lymphoid cells. Mucosal Immunol. (2014) 7:958–68. 10.1038/mi.2013.114 24368564 PMC4165592

[82] Yu X Pappu R Ramirez-Carrozzi V Ota N Caplazi P Zhang J. TNF superfamily member TL1A elicits type 2 innate lymphoid cells at mucosal barriers. Mucosal Immunol. (2014) 7:730–40. 10.1038/mi.2013.92 24220298 PMC3998636

[83] Malhotra N Leyva-Castillo JM Jadhav U Barreiro O Kam C O'Neill NK. RORalpha-expressing T regulatory cells restrain allergic skin inflammation. Sci Immunol. (2018) 3:eaao6923. 10.1126/sciimmunol.aao6923 29500225 PMC5912895

[84] Salimi M Xue L Jolin H Hardman C Cousins DJ McKenzie AN. Group 2 innate lymphoid cells express functional NKp30 receptor inducing type 2 cytokine production. J Immunol. (2016) 196:45–54. 10.4049/jimmunol.1501102 26582946 PMC4913864

[85] Babic M Romagnani C. Boosting type 2 Immunity: when OX40L Comes from ILC2s. Immunity (2018) 48:1067–9. 10.1016/j.immuni.2018.06.004 29924969

[86] Halim TYF Rana BMJ Walker JA Kerscher B Knolle MD Jolin HE. Tissue-restricted adaptive type 2 immunity is orchestrated by expression of the costimulatory molecule ox40l on group 2 innate lymphoid cells. Immunity (2018) 48:1195–207.e1196. 10.1016/j.immuni.2018.05.003 29907525 PMC6015114

[87] van Meer G Voelker DR Feigenson GW. Membrane lipids: where they are and how they behave. Nat Rev Mol Cell Biol. (2008) 9:112–24. 10.1038/nrm2330 18216768 PMC2642958

[88] Haeggstrom JZ Funk CD. Lipoxygenase and leukotriene pathways: biochemistry, biology, and roles in disease. Chem Rev. (2011) 111:5866–98. 10.1021/cr200246d 21936577

[89] Alhouayek M Muccioli GG. COX-2-derived endocannabinoid metabolites as novel inflammatory mediators. Trends Pharmacol Sci. (2014) 35:284–92. 10.1016/j.tips.2014.03.001 24684963

[90] Xue L Salimi M Panse I Mjosberg JM McKenzie AN Spits H. Prostaglandin D2 activates group 2 innate lymphoid cells through chemoattractant receptor-homologous molecule expressed on TH2 cells. J Allergy Clin Immunol. (2014) 133:1184–94. 10.1016/j.jaci.2013.10.056 24388011 PMC3979107

[91] Zhou W Toki S Zhang J Goleniewksa K Newcomb DC Cephus JY. Prostaglandin I2 Signaling and Inhibition of Group 2 Innate Lymphoid Cell Responses. Am J Respir Crit Care Med. (2016) 193:31–42. 10.1164/rccm.201410-1793OC 26378386 PMC4731613

[92] Maric J Ravindran A Mazzurana L Bjorklund AK Van Acker A Rao A. Prostaglandin E2 suppresses human group 2 innate lymphoid cell function. J Allergy Clin Immunol. (2018) 141:1761–73.e1766. 10.1016/j.jaci.2017.09.050 29217133 PMC5929462

[93] Chang JE Doherty TA Baum R Broide D. Prostaglandin D2 regulates human type 2 innate lymphoid cell chemotaxis. J Allergy Clin Immunol. (2014) 133:899–901.e893. 10.1016/j.jaci.2013.09.020 24210841 PMC3943597

[94] Wojno ED Monticelli LA Tran SV Alenghat T Osborne LC Thome JJ. The prostaglandin D(2) receptor CRTH2 regulates accumulation of group 2 innate lymphoid cells in the inflamed lung. Mucosal Immunol. (2015) 8:1313–23. 10.1038/mi.2015.21 25850654 PMC4598246

[95] Doherty TA Khorram N Lund S Mehta AK Croft M Broide DH Lung Type 2 innate lymphoid cells express CysLT1R that regulates Th2 cytokine production. J Allergy Clin Immunol. (2013) 132:205–13. 10.1016/j.jaci.2013.03.048 23688412 PMC3704056

[96] Lund SJ Portillo A Cavagnero K Baum RE Naji LH Badrani JH. Leukotriene C4 potentiates IL-33-induced group 2 innate lymphoid cell activation and lung inflammation. J Immunol. (2017) 199:1096–104. 10.4049/jimmunol.1601569 28667163 PMC5531601

[97] Salimi M Stoger L Liu W Go S Pavord I Klenerman P. Cysteinyl leukotriene E4 activates human group 2 innate lymphoid cells and enhances the effect of prostaglandin D2 and epithelial cytokines. J Allergy Clin Immunol. (2017) 140:1090–100.e1011. 10.1016/j.jaci.2016.12.958 28115217 PMC5624780

[98] Barnig C Levy BD. Innate immunity is a key factor for the resolution of inflammation in asthma. Eur Respir Rev. (2015) 24:141–53. 10.1183/09059180.00012514 25726564 PMC4490858

[99] Barnig C Cernadas M Dutile S Liu X Perrella M Kazani S. Lipoxin A_4_ regulates natural killer cell and type 2 innate lymphoid cell activation in asthma. Sci Transl Med. (2013) 5. 10.1126/scitranslmed.3004812 23447017 PMC3823369

[100] Dustin ML Bivona TG Philips MR. Membranes as messengers in T cell adhesion signaling. Nat Immunol. (2004) 5:363–72. 10.1038/ni1057 15052266

[101] Springer TA. Adhesion receptors of the immune system. Nature (1990) 346:425–34. 10.1038/346425a0 1974032

[102] Mukhopadhyay S Malik P Arora SK Mukherjee TK. Intercellular adhesion molecule-1 as a drug target in asthma and rhinitis. Respirology (2014) 19:508–13. 10.1111/resp.12285 24689994

[103] Karta MR Rosenthal PS Beppu A Vuong CY Miller M Das S. beta2 integrins rather than beta1 integrins mediate Alternaria-induced group 2 innate lymphoid cell trafficking to the lung. J Allergy Clin Immunol. (2018) 141:329–38.e312. 10.1016/j.jaci.2017.03.010 28366795 PMC5623168

[104] Huang Y Mao K Chen X Sun MA Kawabe T Li W. S1P-dependent interorgan trafficking of group 2 innate lymphoid cells supports host defense. Science (2018) 359:114–9. 10.1126/science.aam5809 29302015 PMC6956613

[105] Lei AH Xiao Q Liu GY Shi K Yang Q Li X. ICAM-1 controls development and function of ILC2. J Exp Med. (2018) 215:2157–74. 10.1084/jem.20172359 30049704 PMC6080904

[106] Masuyama K Morishima Y Ishii Y Nomura A Sakamoto T Kimura T. Sputum E-cadherin and asthma severity. J Allergy Clin Immunol. (2003) 112:208–9. 10.1067/mai.2003.1526 12847501

[107] Ganea D. Neuropeptides: Active Participants in Regulation of Immune Responses in the CNS and Periphery. Brain Behav Immun. (2008) 22:33–4. 10.1016/j.bbi.2007.06.014 17706916 PMC2683245

[108] Mishra NC Rir-sima-ah J Boyd RT Singh SP Gundavarapu S Langley RJ. Nicotine inhibits Fc epsilon RI-induced cysteinyl leukotrienes and cytokine production without affecting mast cell degranulation through alpha 7/alpha 9/alpha 10-nicotinic receptors. J Immunol. (2010) 185:588–96. 10.4049/jimmunol.0902227 20505147 PMC2954495

[109] Galle-Treger L Suzuki Y Patel N Sankaranarayanan I Aron JL Maazi H. Nicotinic acetylcholine receptor agonist attenuates ILC2-dependent airway hyperreactivity. Nat Commun. (2016) 7:13202. 10.1038/ncomms13202 27752043 PMC5071851

[110] Moriyama S Brestoff JR Flamar AL Moeller JB Klose CSN Rankin LC. beta2-adrenergic receptor-mediated negative regulation of group 2 innate lymphoid cell responses. Science (2018) 359:1056–61. 10.1126/science.aan4829 29496881

[111] Wallrapp A Riesenfeld SJ Burkett PR Abdulnour RE Nyman J Dionne D. The neuropeptide NMU amplifies ILC2-driven allergic lung inflammation. Nature (2017) 549:351–6. 10.1038/nature24029 28902842 PMC5746044

[112] Townsend EA Miller VM Prakash YS. Sex differences and sex steroids in lung health and disease. Endocr Rev. (2012) 33:1–47. 10.1210/er.2010-0031 22240244 PMC3365843

[113] Cephus JY Stier MT Fuseini H Yung JA Toki S Bloodworth MH. Testosterone Attenuates Group 2 Innate lymphoid cell-mediated airway inflammation. Cell Rep. (2017) 21:2487–99. 10.1016/j.celrep.2017.10.110 29186686 PMC5731254

[114] Kissick HT Sanda MG Dunn LK Pellegrini KL On ST Noel JK. Androgens alter T-cell immunity by inhibiting T-helper 1 differentiation. Proc Natl Acad Sci USA. (2014) 111:9887–92. 10.1073/pnas.1402468111 24958858 PMC4103356

[115] Newcomb DC Cephus JY Boswell MG Fahrenholz JM Langley EW Feldman AS. Estrogen and progesterone decrease let-7f microRNA expression and increase IL-23/IL-23 receptor signaling and IL-17A production in patients with severe asthma. J Allergy Clin Immunol. (2015) 136:1025–34.e1011. 10.1016/j.jaci.2015.05.046 26242299 PMC4600442

[116] Laffont S Blanquart E Savignac M Cenac C Laverny G Metzger D. Androgen signaling negatively controls group 2 innate lymphoid cells. J Exp Med. (2017) 214:1581–92. 10.1084/jem.20161807 28484078 PMC5461006

[117] Warren KJ Sweeter JM Pavlik JA Nelson AJ Devasure JM Dickinson JD. Sex differences in activation of lung-related type 2 innate lymphoid cells in experimental asthma. Ann Allergy Asthma Immunol. (2017) 118:233–4. 10.1016/j.anai.2016.11.011 28017508 PMC5291757

[118] Kadel S Ainsua-Enrich E Hatipoglu I Turner S Singh S Khan S. A Major Population of Functional KLRG1(-) ILC2s in Female Lungs Contributes to a Sex Bias in ILC2 Numbers. Immunohorizons (2018) 2:74–86. 10.4049/immunohorizons.1800008 29568816 PMC5860819

[119] Bartemes K Chen CC Iijima K Drake L Kita H. IL-33-Responsive Group 2 innate lymphoid cells are regulated by female sex hormones in the uterus. J Immunol. (2018) 200:229–36. 10.4049/jimmunol.1602085 29133293 PMC5736420

[120] Ferrara N Gerber HP LeCouter J. The biology of VEGF and its receptors. Nat Med. (2003) 9:669–76. 10.1038/nm0603-669 12778165

[121] Shen X Pasha MA Hidde K Khan A Liang M Guan W. Group 2 innate lymphoid cells promote airway hyperresponsiveness through production of VEGFA. J Allergy Clin Immunol. (2018) 141:1929–31.e1924. 10.1016/j.jaci.2018.01.005 29382598 PMC6019125

[122] Humbles AA Lu B Nilsson CA Lilly C Israel E Fujiwara Y. A role for the C3a anaphylatoxin receptor in the effector phase of asthma. Nature (2000) 406:998–1001. 10.1038/35023175 10984054

[123] Krug N Tschernig T Erpenbeck VJ Hohlfeld JM Kohl J. Complement factors C3a and C5a are increased in bronchoalveolar lavage fluid after segmental allergen provocation in subjects with asthma. Am J Respir Crit Care Med. (2001) 164:1841–3. 10.1164/ajrccm.164.10.2010096 11734433

[124] Gasteiger G Fan X Dikiy S Lee SY Rudensky AY. Tissue residency of innate lymphoid cells in lymphoid and nonlymphoid organs. Science (2015) 350:981–5. 10.1126/science.aac9593 26472762 PMC4720139

